# Localization of EccA_3_ at the growing pole in *Mycobacterium smegmatis*

**DOI:** 10.1186/s12866-022-02554-6

**Published:** 2022-05-19

**Authors:** Nastassja L. Kriel, Mae Newton-Foot, Owen T. Bennion, Bree B. Aldridge, Carolina Mehaffy, John T. Belisle, Gerhard Walzl, Robin M. Warren, Samantha L. Sampson, Nico C. Gey van Pittius

**Affiliations:** 1 DSI-NRF Centre of Excellence for Biomedical Tuberculosis Research; South African Medical Research Council Centre for Tuberculosis Research; Division of Molecular Biology and Human Genetics, Faculty of Medicine and Health Sciences, Stellenbosch University, Cape Town, South Africa; 2 Department of Molecular Biology and Microbiology, Tufts University School of Medicine, Boston, MA 02111 USA; 3 Mycobacteria Research Laboratories, Department of Microbiology Immunology and Pathology, Colorado State University, Fort Collins, CO 80523 USA

**Keywords:** ESX-3, Type VII secretion, Mycobacterium, Polar localization, EccA_3_

## Abstract

**Background:**

Bacteria require specialized secretion systems for the export of molecules into the extracellular space to modify their environment and scavenge for nutrients. The ESX-3 secretion system is required by mycobacteria for iron homeostasis. The ESX-3 operon encodes for one cytoplasmic component (EccA_3_) and five membrane components (EccB3 – EccE3 and MycP_3_). In this study we sought to identify the sub-cellular location of EccA_3_ of the ESX-3 secretion system in mycobacteria.

**Results:**

Fluorescently tagged EccA_3_ localized to a single pole in the majority of *Mycobacterium smegmatis* cells and time-lapse fluorescent microscopy identified this pole as the growing pole. Deletion of ESX-3 did not prevent polar localization of fluorescently tagged EccA_3_, suggesting that EccA_3_ unipolar localization is independent of other ESX-3 components. Affinity purification - mass spectrometry was used to identify EccA_3_ associated proteins which may contribute to the localization of EccA_3_ at the growing pole. EccA_3_ co-purified with fatty acid metabolism proteins (FAS, FadA3, KasA and KasB), mycolic acid synthesis proteins (UmaA, CmaA1), cell division proteins (FtsE and FtsZ), and cell shape and cell cycle proteins (MurS, CwsA and Wag31). Secretion system related proteins Ffh, SecA1, EccA1, and EspI were also identified.

**Conclusions:**

Time-lapse microscopy demonstrated that EccA3 is located at the growing pole in *M. smegmatis*. The co-purification of EccA_3_ with proteins known to be required for polar growth, mycolic acid synthesis, the Sec secretion system (SecA1), and the signal recognition particle pathway (Ffh) also suggests that EccA_3_ is located at the site of active cell growth.

**Supplementary Information:**

The online version contains supplementary material available at 10.1186/s12866-022-02554-6.

## Introduction

Type VII secretion systems are encoded by mycobacteria and various other organisms belonging to phylum Actinobacteria [1]. In mycobacteria, these secretion systems are also referred to as ESAT-6 or ESX secretion systems. The *Mycobacterium tuberculosis* genome contains five ESX gene cluster regions, which encode five ESX secretion systems, ESX-1 to ESX-5 [1]. The well-studied ESX-1- secretion system contributes to virulence of pathogenic strains through the secretion of ESAT-6, CFP-10 and the ESX-1 secretion-associated proteins (Esp) [2–4]. Likewise, ESX-3 and ESX-5 also facilitate pathogenesis through the secretion of Esx proteins as well as members of the proline-glutamic acid (PE) and proline-proline-glutamic acid (PPE) protein family (characterised by their Proline and Glutamic acid N-terminal motifs) [5–7]. *M. smegmatis* also encodes three ESX secretion systems, ESX-1, −3 and − 4, and although ESX-1 and ESX-3 have been linked to bacterial virulence in *M. tuberculosis*, the same cannot be said for *M. smegmatis*. *M. smegmatis* is considered a non-pathogenic organism, however documented cases of skin, soft-tissue or infection of immunocompromised individuals have previously been reported [8, 9]. *M. smegmatisM. smegmatisM. smegmatis*ESX-3 is required for *M. tuberculosis* growth through mycobactin-mediated iron acquisition, and deletions can only be tolerated in the presence of iron supplemented culture media [6, 10, 11]. Although the paralogue *M. smegmatis* ESX-3 secretion system does not contribute to pulmonary tuberculosis infections, it has been shown to functionally complement the *in vitro* iron-related growth defects in a *M. tuberculosis* ESX-3 knock-out strain (ΔESX-3_MTB_) [6]. This functional complementation of ΔESX-3_MTB_ by the *M. smegmatis* ESX-3 secretion system suggests that the iron homeostasis functionality of ESX-3 is conserved between species. The conserved functionality of ESX-3 across species suggests that *M. smegmatis* may serve as an appropriate model organism for investigating the location and functionality of the ESX-3 secretion system. *M. smegmatis.*

Cellular components and macromolecular complexes required for various cellular processes, including DNA replication and cell division display sub-cellular arrangements within bacterial cells [12, 13]. Mycobacteria exhibit asymmetrical polar growth and division, resulting in a heterogeneous population of cells [14–16]. Several studies demonstrate the localization of peptidoglycan, arabinogalactan, and mycolic acid synthesis at the mycobacterial poles [16, 17]. Interestingly, components of the ESX-1 secretion system also localize with these cellular processes in *Mycobacterium marinum* and *M. smegmatis* [12, 18]. An EccCa_1_ homologue from *M. marinum*, Mh3870, localizes to the poles, showing a preference for “new” poles (the septal pole) with peptidoglycan synthesis, suggesting that ESX-1 is located at the site of active cell growth in *M. marinum* [18]. In *M. smegmatis*, EccCb_1_ (MSMEG_0062) and *M. tuberculosis* EccCb_1_ (Rv3871), localize at the “old” pole (the pole distal to the septum), previously identified as the growing pole in *M. smegmatis* [12, 14]. The localization of a *M. tuberculosis* homologue ESX-1 protein at the growing pole in *M. smegmatis* suggests that the translocation signal to the growing pole is conserved across species [12]. Although these results are conflicting as to whether ESX-1 can be associated with the “old” or “new” pole, a common theme is the localization of ESX-1 components at the position of active cell growth with a preference for a single mycobacterial pole.

Conserved components of the ESX-3 secretion system include five membrane components, EccB_3_ – EccE_3_ and MycP_3_, as well as one cytosolic component, EccA_3_ [1, 19]. In *M. tuberculosis*, both EccA_3_ and EccA_1_ have been shown to exist as hexamers in the presence of ATP [20, 21]. The C-terminal region of both these proteins includes a CbbX-like AAA-domain which exhibits ATPase activity and also acts as an oligomerization domain. The “open-close” movements of the N-terminal domain upon ATP-binding by the C-terminal domain have been suggested to facilitate EccA interaction with other components of the secretion pathway, most notably secretion substrates of the ESX-3 secretion system [20, 21].

In this study we sought to identify the sub-cellular location of the ESX-3 component, EccA_3_ (MSMEG_0615), in *M. smegmatis*. We hypothesized that EccA_3_ may localize to the growing pole in *M. smegmatis*, as previously shown for components of the ESX-1 secretion system [12]. The localisation of EccA_3_ at the growing polar region of *M. smegmatis* along with the extensive data for ESX-1 localization would suggest a conserved mechanism whereby ESX secretion systems are recruited to the growing pole of the mycobacterial membrane. To identify possible EccA_3_ interacting proteins which may contribute to unipolar localization at the growing pole, we performed affinity purification followed by mass spectrometry (AP-MS). Localization of both ESX-1 and ESX-3 components at the growing pole may suggest a conserved mechanism by which these secretion systems are incorporated into the membrane at the growing pole.

## Results

### EccA_3_ from ESX-3 localizes at a single pole in *M. smegmatis*

**Fig. 1:**
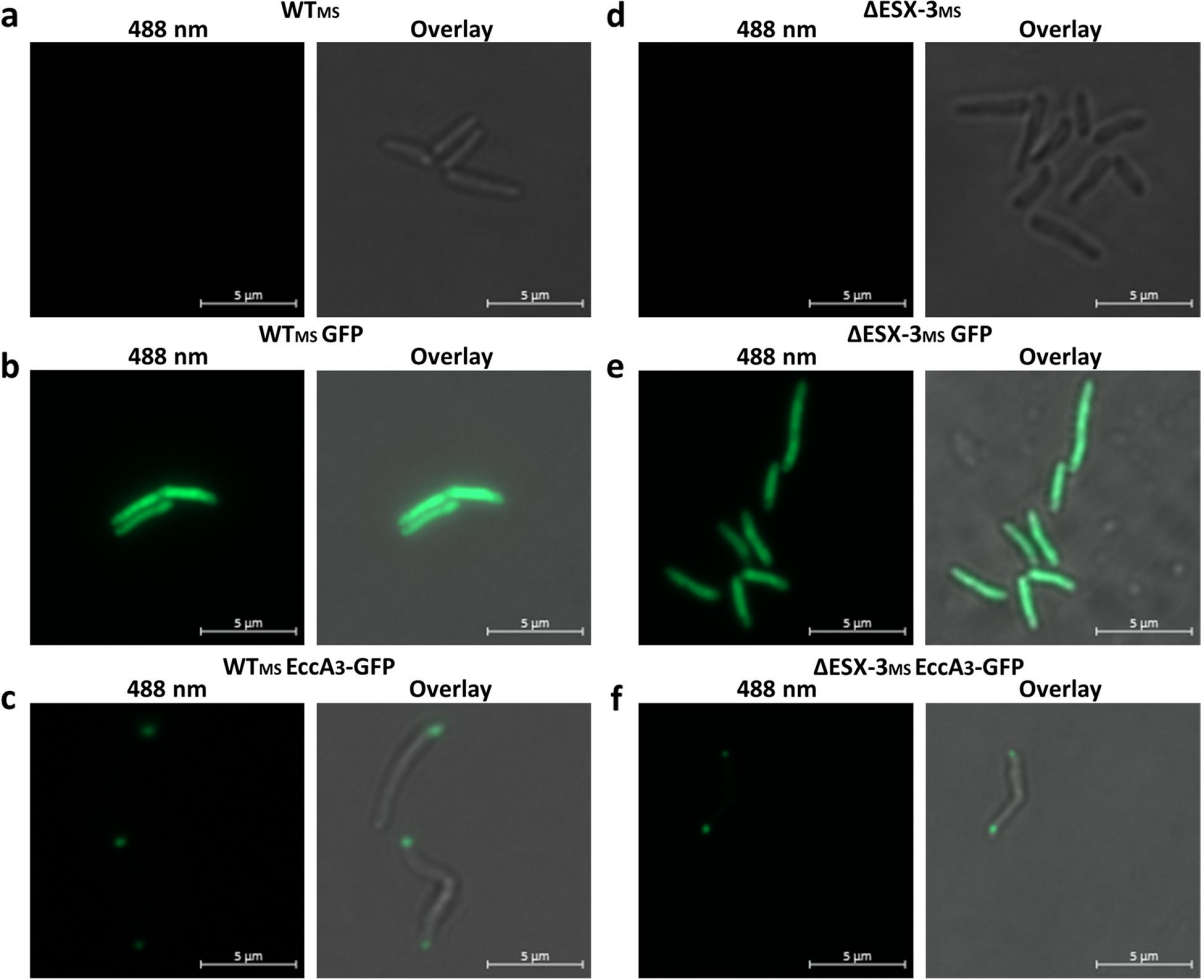
Unipolar localization of EccA_3_ in *Mycobacterium smegmatis*. Fluorescent microscopy demonstrated no GFP fluorescence of WT_MS_ (**a**) and ΔESX-3_MS_ (**d**) cells. GFP fluorescence was detected throughout the cell in positive control WT_MS_::pDMNI (**b**) and ΔESX-3_MS_::pDMNI (**e**) *M. smegmatis*. EccA_3_ was found to reside at a single pole within WT_MS_::pDMNI0615 (**c**) and ΔESX-3_MS_::pDMNI0615 (**f**) cells

**Fig. 2:**
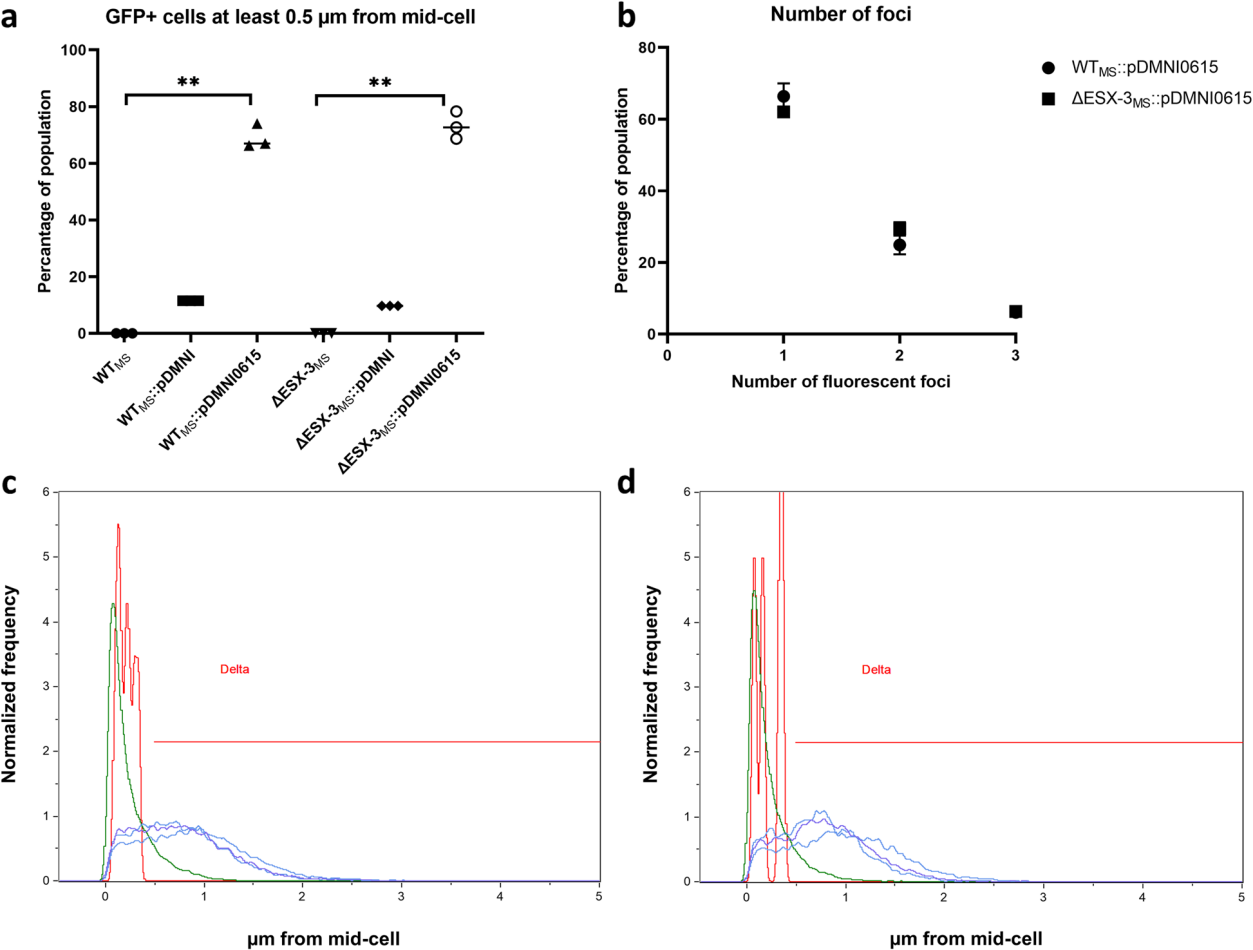
Localization of EccA_3_ within a population of *Mycobacterium smegmatis*. Three independent clonal populations were used for imaging flow cytometry experiments. **a** Imaging flow cytometry was used to demonstrate that more than 60% of clonal populations of WT_MS_::pDMNI0615 and ΔESX-3_MS_::pDMNI0615 had a GFP focus at least 0.5 μm from the mid-cell when compared to no GFP (WT_MS_ and ΔESX-3_MS_) and GFP positive controls (WT_MS_::pDMNI and ΔESX-3_MS_::pDMNI). Unipolar localisation was significant following a Kruskal-Wallis test with a p-value of 0.0036 for both WT_MS_ and ΔESX-3_MS_ (indicated with **). **b** The majority of GFP positive WT_MS_::pDMNI0615 and ΔESX-3_MS_::pDMNI0615 cells with a fluorescent focus at least 0.5 μm from the mid-cell had only one fluorescent focus. **c** The histogram shows distance of a fluorescent foci from the mid-cell of populations WT_MS_ (red), WT_MS_::pDMNI (green), WT_MS_::pDMNI0615.1 (blue), WT_MS_::pDMNI0615.2 (blue-purple), WT_MS_::pDMNI0615.3 (purple). **d** The distance of fluorescent foci from the mid-cell of ΔESX-3_MS_ (red), ΔESX-3_MS_::pDMNI (green), ΔESX-3_MS_::pDMNI0615.1 (blue), ΔESX-3_MS_::pDMNI0615.2 (blue-purple), ΔESX-3_MS_::pDMNI0615.3 (purple) populations. A population with GFP fluorescence located at least 0.5 μm from the mid-cell, represented by the red line called Delta, was used to score the number of fluorescent foci per cell

To determine the sub-cellular location of EccA_3_ in *M. smegmatis*, we constitutively expressed EccA_3_ as a C-terminal fluorescently tagged protein under the control of a *psmyc* promoter (native to *M. smegmatis*). To reduce steric interference between the green fluorescent protein (GFP) and the C-terminal oligomerization domain, a 10 amino acid linker (ASGSAGSAGSA) was used. Control experiments revealed that untransformed *M. smegmatis* mc^2^155 (WT_MS_) displayed no fluorescence (Fig. 1A), whereas expression of recombinant GFP resulted in distributed fluorescence throughout WT_MS_ (Fig. 1B). Expression of EccA_3_-GFP in WT_MS_ resulted in fluorescence at a single polar region *M. smegmatis* cells (Fig. 1C). Imaging flow cytometry demonstrated that the GFP signal of EccA_3_-GFP expressing cells was located at least 0.5 μm from the mid-cell for majority of the population of three independent clonal populations of WT_MS_::pDMNI0615 (Fig. 2A, C, Table S4). Of the cells which displayed a fluorescent signal at least 0.5 μm from the mid-cell, the majority had a single fluorescent focus (Fig. 2B, Table S4). These results suggest that EccA_3_ is located at a single polar region in *M. smegmatis*, similar to what was observed for EccCb_1_ from ESX-1 [12].

We expressed EccA_3_-GFP in a *M. smegmatis* mc^2^155 ESX-3 knock-out strain (ΔESX-3_MS_) mutant strain to determine if unipolar localization of EccA_3_-GFP is dependent on any other proteins encoded by the ESX-3 gene cluster. Control strains demonstrated no GFP fluorescence for untransformed ΔESX-3_MS_ (Fig. 1D), whereas constitutively expressed GFP demonstrated fluorescence throughout the deletion mutant (Fig. 1E). Similar to WT_MS_, EccA_3_-GFP demonstrated unipolar localization in ΔESX-3_MS_ cells (Fig. 1F). Likewise, imaging flow cytometry showed that the GFP signal in EccA_3_-GFP expressing cells of three independent clonal populations of ΔESX-3_MS_::pDMNI0615 was predominantly located at least 0.5 μm from the mid-cell (Fig. 2A, D, Table S4). A single GFP focus was detected within the majority of cells in which EccA_3_-GFP was located at least 0.5 μm from the mid-cell (Fig. 2B, Table S4). These results suggest that EccA_3_ localization is independent of other components of the ESX-3 secretion system.

### EccA_3_ from ESX-3 localizes at a growing pole in *M. smegmatis*

**Fig. 3:**
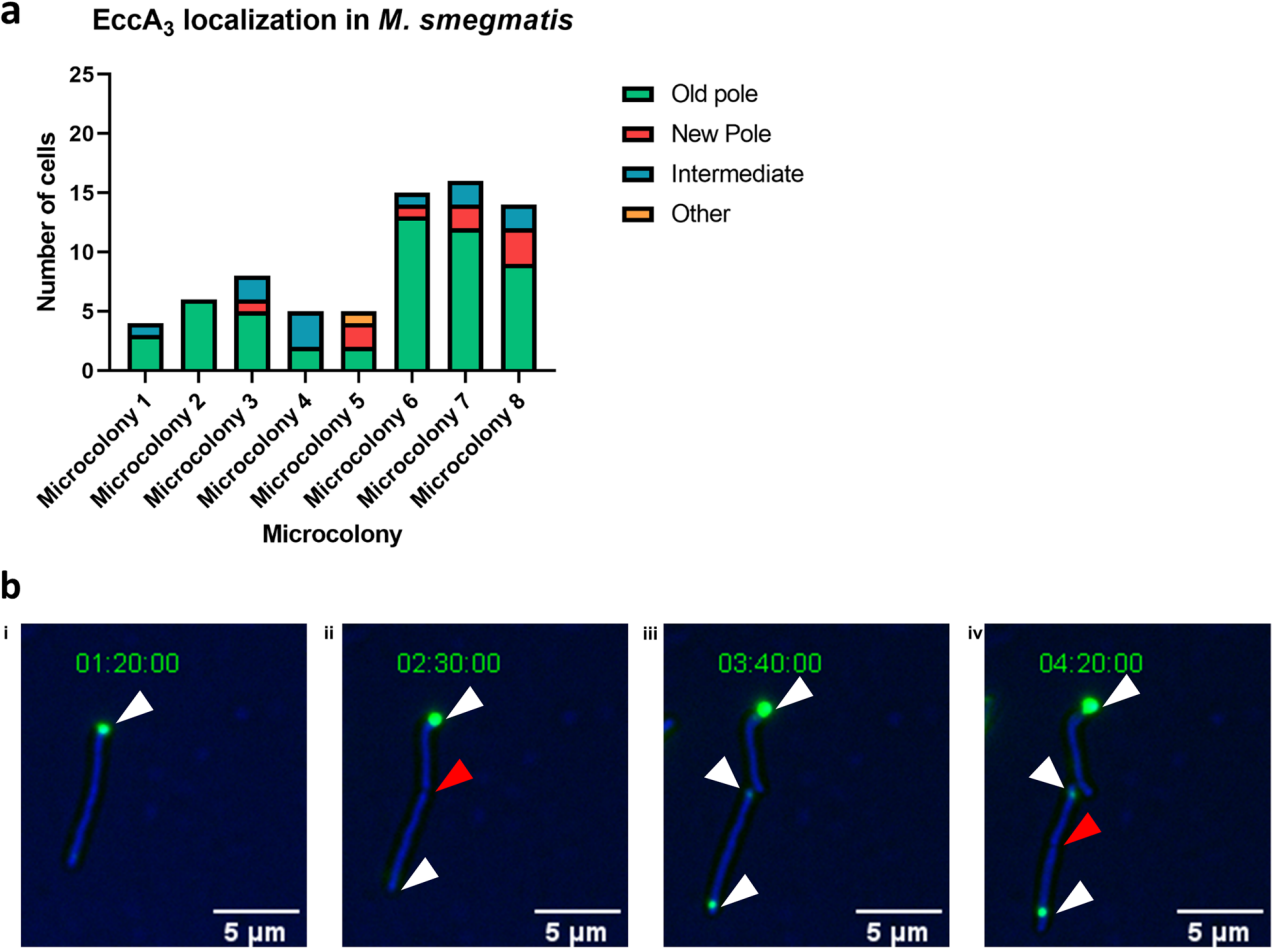
EccA_3_ localizes at the growing pole in *Mycobacterium smegmatis*. **a** Time-lapse imaging of eight independent clonal populations demonstrated that EccA_3_ localized at the old pole in 52 of the 73 cells imaged. **b** A single WT_MS_::pDMNI0615 cell with a fluorescent focus at the pole (i, white arrow) can be seen dividing into two cells (ii) with a red arrow at the septum. A second fluorescent foci representing EccA_3_-GFP can be seen at the old pole (iii, white arrow) before a third fluorescent foci (iv, white arrow) is detected following cell division (iv, red arrow at the septum). One of the fluorescent foci detected previously at the old pole (iii, white arrow) migrated to be polar adjacent (iv, white arrow)

The ESX-1 secretion system was suggested to be located at the non-septal pole (“old pole”) which is the faster growing pole of asymmetrically growing *M. smegmatis* [12, 14]. To investigate whether EccA_3_ from ESX-3 was also located at the non-septal pole of *M. smegmatis*, time-lapse fluorescent microscopy imaging of eight independent clonal populations of *M. smegmatis* expressing EccA_3_-GFP was performed (Fig. 3).

In agreement with fluorescence microscopy and imaging flow cytometry, time-lapse imaging demonstrated that EccA_3_ localized to a single pole in *M. smegmatis*. Of the 73 cells that had undergone cell division from eight independent clonal populations, 71% (52 cells) showed polar localization of EccA_3_-GFP at the non-septal pole in *M. smegmatis* (Fig. 3A). Of the 29% (21 cells) that did not show localization at the “old pole”, 12% (9 cells) had foci at the “new pole”, 15% (11 cells) had foci in mid-cell positions, and 1% (1 cell) could not be scored (Fig. 3A). Figure 3B follows the division of one representative cell into three, with the appearance of new fluorescent foci representing EccA_3_-GFP at the poles. Initially a single focus is seen at a pole (Fig. 3 Bi) which remains associated with that pole following cell division at the septum (red arrow) (Fig. 3 Bii). New fluorescent foci appear at the old poles (the inherited pole) of daughter cells following cell division (white arrow) (Fig. 3 Biii). One new fluorescent foci does appear to not stay at the pole (Fig. 3 Biv), but remains adjacent to the pole. Together, these data demonstrated that EccA_3_ from ESX-3 predominantly localized to the growing non-septal pole of *M. smegmatis*.

### EccA_3_ co-purifies with proteins from the sec secretion system

To identify EccA_3_ associated proteins, we affinity purified N-terminally FLAG-tagged EccA_3_ from formaldehyde treated WT_MS_ using an anti-FLAG antibody immobilized on protein G magnetic beads We identified 5156 unique peptides which mapped to 270 protein groups with at least two unique peptides each. Principal component analysis revealed separate clustering of anti-FLAG- EccA_3_ (Immunoprecipitations) IPs from control IPs (Fig. S3). The 270 identified protein groups mapped to 269 MSMEG annotations and included 216 high confidence proteins (with no protein identifications made within any of the control IPs) and 53 low confidence proteins (with protein identifications in control IPs but statistically significantly more abundant in anti-FLAG-EccA_3_ IPs) (Table S5-S7). Bioinformatics analysis using Database for Annotation, Visualization and Integrated Discovery (DAVID) revealed an enrichment for Gene Ontology (GO) terms associated with translation (biological processes), structural constituent of ribosome (molecular function) and ribosome (cellular component) (Table S9) [22]. Other highly enriched GO terms included tricarboxylic acid cycle and ATP binding, which are likely from proteins required to provide energy for the translational processes (Table S9).

Possible EccA_3_ interacting proteins with the highest relative abundance are described in Table 1. Notably, several of the proteins identified with the highest abundance are involved in translation and protein folding (EF-Tu, GroEL1, GroEL2, GroES and DnaK). Interestingly, Fatty acid synthase (FAS), and another fatty acid metabolism protein FadA3 were also identified in high abundance (Table 1). Less abundant proteins with gene ontologies related to cell division (FtsE and FtsZ), cell shape and cell cycle (MurS, CwsA and Wag31), fatty acid (KasA and KasB), mycolic acid (UmaA, CmaA1), and secretion systems (Ffh from the SRP pathway, SecA1 from the Sec secretion system, and EccA1 and EpsI from the ESX-1 secretion system) were also identified (Table S5).

**Table 1:** Highest abundance proteins identified using AP-MS

Uniprot Annotation	MSMEG Annotation	RV homologue	Gene Name	Protein Names	Gene Ontology	IFQ Intensity
A0QS98	MSMEG_1401	Rv0685	*tuf*	Elongation factor Tu (EF-Tu)	cytoplasm; GTPase activity; GTP binding; translation elongation factor activity	1,509,500,000
A0QQU5	MSMEG_0880	Rv0440	*groL1*	60 kDa chaperonin 1 (GroEL protein 1)	ATP binding; cytoplasm; protein refolding	464,740,000
A0R729	MSMEG_6759		*glpK*	Glycerol kinase	ATP binding; glycerol-3-phosphate metabolic process; glycerol catabolic process; glycerol kinase activity	257,030,000
A0R2Y1	MSMEG_5273	Rv1074c	*fadA3*	Acetyl-CoA acetyltransferase	transferase activity, transferring acyl groups other than amino-acyl groups	206,560,000
A0QSS4	MSMEG_1583	Rv3417c	*groL2*	60 kDa chaperonin 2 (GroEL protein 2)	ATP binding; cytoplasm; protein refolding	198,490,000
A0QWW2	MSMEG_3084	Rv1436	*gap*	Glyceraldehyde-3-phosphate dehydrogenase	cytoplasm; glucose metabolic process; glyceraldehyde-3-phosphate dehydrogenase (NAD+) (phosphorylating) activity; glycolytic process; NAD binding; NADP binding	189,420,000
A0QUX8	MSMEG_2374	Rv3001c	*ilvC*	Ketol-acid reductoisomerase	coenzyme binding; isoleucine biosynthetic process; ketol-acid reductoisomerase activity; valine biosynthetic process	183,580,000
A0QQC8	MSMEG_0709	Rv0350	*dnaK*	Chaperone protein DnaK	ATP binding; protein folding	141,160,000
A0QSS3	MSMEG_1582	Rv3418c	*groS*	10 kDa chaperonin (GroES protein)	ATP binding; cytoplasm; protein folding	132,270,000
A0R1H7	MSMEG_4757	Rv2524c	*fas*	Fatty acid synthase	enoyl-[acyl-carrier-protein] reductase (NADH) activity; fatty acid biosynthetic process; fatty acid synthase complex; nucleotide binding	101,460,000

## Discussion

Bacteria require specialized secretion systems to export molecules into the extracellular space to modify their environment and scavenge for nutrients to survive and propagate. Several secretion systems in rod-shaped bacteria localize at the bacterial poles, including the type II secretion system in *Vibrio cholerae*, the type III secretion system in *Salmonella*, the type IV secretion system in *Coxiella burnetti* and *Legionella pneumophila*, and the type V secretion system in *Shigella flexneri* [23–27]. Likewise, in mycobacteria, components of the ESX-1 secretion systems in both *M. marinum* and *M. smegmatis* have shown unipolar localization [12, 18]. The reason for the polar localization of these secretion systems remains unknown and may differ between the various secretion systems and organisms. However, site-directed secretion of substrates may result in a crucial concentration of effector proteins or substrates for bacteria-environmental reactions. The polar localization of the type IV secretion system in *L. pneumophila* was recently demonstrated to contribute to virulence through its ability to alter the host’s endocytic pathway [26]. The authors speculated that the polar localization of the type IV secretion system contributed to the pathogenesis of the organism either by effector protein concentration, the hierarchical display of effector proteins or spatial restriction imposed by the Legionella containing vacuole [26]. Polar localization of the Type VI secretion system in *Francisella novicida* and *Burkholderia thailandensis* has also been suggested to increase effector delivery and/or the coordination of other polarly localized complexes, such as pili, for protein translocation through the Type VI secretion system [28–30].

In this study, we sought to identify the subcellular location of EccA_3_ in *M. smegmatis*. We elected to fluorescently tag the C-terminal of EccA_3_ with a linker chain to reduce steric hindrance and to limit interference with the suggested functional N-terminal domain. Regardless of the inclusion of a linker, a 29 kDa GFP tag may still interfere with protein function or protein-protein interactions. Fluorescent microscopy and imaging flow cytometry analysis was used to show that C-terminal GFP tagged EccA_3_ localized to a single pole in WT_MS_ (Figs. 1, and 2). Time-lapse microscopy demonstrated that EccA_3_ localizes at the growing polar region of the *M. smegmatis* bacilli (Fig. 3), suggesting that the ESX-3 secretion system is either located at the growing pole or may be incorporated into the growing mycobacterial membrane at the old pole.

We next sought to explore how EccA_3_ localizes at the old pole in *M. smegmatis*. The translocation of proteins to the bacterial pole is often mediated through interactions with other proteins, also referred to as the diffusion-and-capture mechanism [13]. Other suggested mechanisms include the negative curvature mechanism, the nucleoid occlusion mechanism and the affinity for polar features of the cell envelope mechanism [13]. We hypothesized that EccA_3_ may interact with other protein(s) which could facilitate its translocation to the growing pole, or which may already be present at growing pole. We used formaldehyde to stabilize protein-protein interactions followed by AP-MS to target FLAG-tagged EccA_3_. Formaldehyde crosslinking can also crosslink closely associated proteins and may not provide direct evidence of protein-protein interaction, but rather a close association. Highly abundant cellular proteins may serve as protein contaminants in such an assay. Furthermore, formaldehyde crosslinking may result in chemical modification of proteins, limiting our ability to identify all immunoprecipitated proteins using automated database searching.

GO enrichment suggested that proteins with GO terms translation, ribosome and structural constituent of ribosome were most enriched in our assay. Interestingly, chromatin immunoprecipitation followed by sequencing (ChIP-seq) and RNA-seq experiments have previously shown the ESX-3 locus (EccA_3_ to EccE_3_) to be one of the largest transcriptional units in *M. tuberculosis* [31]. The enrichment proteins with GO terms tricarboxylic acid cycle and ATP binding are likely required to provide energy for protein translation and the translocation of proteins to the membrane. We also identified a number of proteins associated with cell division, cell cycle and cell shape (FtsE, FtsZ, MurA, CwsA and Wag31). Wag31 is known to be required for polar growth and the maintenance of cell morphology [32–35] and has also been shown to interact with other proteins required for cell wall synthesis and division. These include AccA3 (also identified in this study, Table S5), a component of Acyl-CoA carboxylase (ACCase) which is required for lipid and mycolic acid synthesis, and the cell wall synthesis protein CwsA [36–38]. Interestingly, the polar localization of the type IV secretion system was also linked to cell division in *L. pneumophila* [39]. The co-purification of EccA_3_ and polar proteins associated with polar growth (Wag31, AccA3 and ParB), was not unexpected as EccA_3_ as well as proteins from ESX-1 localized to the site of active cell growth [12, 18, 37, 40, 41]. Mycolic acid synthesis has been reported to be localized at both the growing pole and the septum, and in *M. marinum*, EccA_1_ was reported to be required for optimal mycolic acid synthesis [42, 43]. EccA_3_ co-purified with mycolic acid synthesis proteins UmaA and CmaA1 (Table S5) as well as fatty acid metabolism proteins FAS, FadA3, AcpM, FadB, FadB2, Des, DesA2, and FabD (Table 1, TableS5). These results, together with the localization of EccA_3_ at the growing pole, suggest that EccA_3_ may also be associated with mycolic acid synthesis at the growing pole in *M. smegmatis*.

AP-MS experiments also identified ESX-1 proteins EccA_1_ and EspI as being associated with EccA_3_, suggesting the co-localization of ESX-1 and ESX-3 components at the growing pole of *M. smegmatis* [12]. Both EccA_1_ and EccA_3_ have previously been reported to form hexamers [20, 21]. It is tempting to speculate that these proteins may hetero-multimerize, however, further experimental analysis is required to investigate this. We identified SecA1 from the Sec secretion pathway (Table S5) as well as Ffh, a GTPase component of the signal recognition pathway (SRP) pathway [44]. The Sec secretion pathway is required for transport over the cytoplasmic membrane and the insertion of integral membrane proteins through ribosome docking via the signal recognition particle (SRP) pathway [44, 45]. Association of EccA_3_ with components of the Sec and SRP pathways may suggest that ESX-3 is incorporated into the mycobacterial membrane through the Sec and SRP pathways. Unfortunately, mass spectrometry did not identify any structural components of the ESX-3 secretion system. AP-MS may have failed to identify these proteins because of their native level of expression, partial hydrophobic surfaces, and their association with membrane lipids. The identification of low abundance proteins is complicated by the large dynamic range and complexity of proteomic experiments, resulting in the oversampling of abundant proteins and the under-detection of low abundance proteins [46]. In the future, associations between EccA_3_ and other ESX-3 components, EccB_3_ - EccE_3_ could be investigated using highly sensitive targeted mass spectrometry approaches, such as selected reaction monitoring (SRM) or multiple reaction monitoring (MRM) methodologies [47]. Additionally, further sub-cellular fractionation of the cytosol, membrane and cell wall components, would also aid in identifying high probability EccA_3_ interacting proteins required for localization.

The co-localization of EccA_3_ together with proteins required for asymmetrical growth and components of the Sec and SRP pathways suggests other components of the ESX-3 secretion system may also be recruited to the growing pole in *M. smegmatis*. The co-localisation of EccA_3_ at the growing pole with components of the ESX-1 secretion system suggest that the process required for localization at the nascent membrane may be conserved for the ESX secretion systems. It has previously been speculated that the polar localization of ESX-1 may be required for site-directed secretion of effector proteins, specifically those required for DNA transfer in *M. smegmatis* [12]. The ESX-3 secretion system is known to be required for mycobactin mediated iron acquisition and a single location within the mycobacterial membrane might limit the efficiency of iron retrieval. Although EccA_3_ localises to the growing pole in *M. smegmatis*, we hypothesize that this may not be the final location of ESX-3, but rather the insertion site. Fluorescent tagging of ESX-3 membrane components and correlative light and electron microscopy experiments may identify the final location of ESX-3 and pinpoint the location of these components within the mycobacterial membrane.

## Methods

### Bacterial strains and growth conditions

*Escherichia coli* XL-1 Blue (Stratagene) (Table S1) was used to propagate plasmid DNA constructs. *E. coli* was cultured in liquid Luria-Bertani (LB) broth with shaking or on LB agar plates at 37 °C supplemented with antibiotics at the following concentrations, as appropriate: ampicillin 100 μg/ml, kanamycin 50 μg/ml or hygromycin 150 μg/ml. For the expression of fluorescently tagged proteins we made use of *M. smegmatis* mc^2^155 (WT_MS_) and a *M. smegmatis* mc^2^155 ESX-3 knock-out strain (ΔESX-3_MS_) (Table S1) [48]. N-terminally FLAG-tagged EccA_3_ was expressed in *M. smegmatis* mc^2^155 for AP-MS experiments. *M. smegmatis* was cultured in Difco™ Middlebrook 7H9 Broth or on BBL™ 7H11 base plates supplemented with 0.5% glucose, 0.02% Tween-80 and 0.5% glycerol at 37 °C. Culture media was supplemented with kanamycin (25 μg/ml) and hygromycin (50 μg/ml) where appropriate (Table S2).

### Plasmid construction

Full length MSMEG_0615 (*ecc*A_3_) was Polymerase Chain Reaction (PCR) amplified from *M. smegmatis* mc^2^155 using Phusion Polymerase Taq (New England Biolabs Inc.) and oligonucleotides described in Table S3. PCR amplified *eccA*_*3*_ was cloned into the commercial plasmid CloneJet (ThermoFisher Scientific) prior to subcloning into pDMNI at restriction sites EcoRI and HindIII, in frame with 3′ *gfp* to create pDMNI0615. To identify possible interacting proteins of EccA_3_ the plasmid pNFLAG0615 was constructed as described [49]. Gene sequence integrity of all plasmid constructs was verified using Sanger sequencing at the Central Analytical Facilities (CAF), Stellenbosch University, South Africa.

### Protein expression

Plasmids required for the localization of GFP (pDMNI) and EccA_3_-GFP (pDMNI0615) were transformed into WT_MS_ and ΔESX-3_MS_ to generate strains described in Table S2. Protein expression was confirmed through immunoblotting using a mouse derived anti-GFP antibody (F56-6A1, Santa Cruz) and a goat anti-mouse horseradish peroxidase conjugated antibody (HAF007, R&D systems) (Fig. S1). The primary and secondary antibodies were used at the final titres of 1:2000 and 1:10,000, respectively.

Plasmids constructed for the purification of FLAG (pNFLAG) and FLAG-MSMEG_0615 (pNFLAG0615) were transformed into WT_MS_ for the purification of possible interacting proteins to generate strains described in Table S2. Protein expression was confirmed using immunoblotting with a mouse derived anti-FLAG antibody (FG4R, ThemoFisher Scientific) and a goat anti-mouse horseradish peroxidase conjugated antibody (HAF007, R&D systems) (Fig. S2). The FG4R antibody was used at a final titre of 1:5000.

### Microscopy and data analysis

*M. smegmatis* strains and transformants (Table S2) were cultured for static imaging of live cells using fluorescent microscopy on a Zeiss AxioObserver wide field microscope using ZEN 2.5 software. Images were acquired using a 63x oil immersion objective. A Colibri 7 LED light source was used at an excitation of 488 nm for fluorescent images and emission was detected from 501–527 nm. Fluorescent and brightfield images were simultaneously acquired with the Axiocam 503 camera. Microscopy data were processed for publication using Zen 2.3 blue edition (Zeiss).

### Imaging flow cytometer analysis

Cultured *M. smegmatis* strains and transformants were sonicated for 12 minutes at 36 kHz (Zues, Sonicator bath) before filtering through a 40 μm filter. Cells were fixed with 4% formaldehyde in phosphate buffered saline (PBS) with 0.05% Tween-80 for 30 minutes. Cells were resuspended in 1000 μL PBS prior to imaging flow cytometer analysis using the Amnis® ImageStream®X Mark II Imaging Flow Cytometer. A minimum of 10,000 in focus GFP events were captured per sample and the data obtained from these experiments were analysed using IDEAS 6.2 software. Signals from bright field and GFP were collected in channels 9 and 2, respectively. The population of focussed and single cells was selected followed by the selection of GFP positive cells using fluorescence intensity. Cells not selected were excluded from downstream analysis because these cells were clumps of cells, not in focus or with low GFP fluorescence intensity. Localization of EccA_3_ at the poles were scored using the adaptive erode mask function with a coefficient of 86 and the delta centroid XY function with intensity weighting on the GFP signal. Cells with a GFP signal at least 0.5 μm from the middle of the cell was selected before counting the number of fluorescent foci using the peak mask (spot to cell background ratio of 1.5) and range mask (fluorescent foci must be at least 0.5 μm) function together with the spot counting feature.

### Time-lapse microscopy

Time-lapse microscopy was performed in a custom flow-cell microfluidic device made of patterned PDMS-fused cover glass that ensured cells remain in two dimensions but in a single focal plane as described in [14, 50]. *M. smegmatis* cells were grown in shallow channels that are continuously supplied with fresh medium throughout the experiment. Bright-field and fluorescent images were acquired every 10 minutes using a DeltaVision PersonalDV microscope with a heated chamber and a 60X objective. Images were captured every 10 minutes to observe and to annotate cell division and fluorescent-foci localization as being either old pole, new pole, mid-cell, or other.

### Immunoprecipitation of FLAG-tagged MSMEG_0615

*M. smegmatis* strains were cultured to an OD_600_ of 0.4 prior to crosslinking with formaldehyde at a final concentration of 1% for 10 min at 37 °C. Cells were washed with PBS prior to being stored at −80 °C. Thawed cell pellets were resuspended in lysis buffer (50 mM Tris-HCl pH 7.5, 150 mM NaCl, 1% Triton X-100) supplemented with protease inhibitors (Roche cOmplete™ mini EDTA-free protease inhibitor cocktail). Cells were sonicated (QSonica Q700 probe sonicator) four times for 20 sec at an amplitude of 30 with 5 min intervals on ice. Protein concentrations of whole cell lysates were determined using a Direct Detect Spectrometer (Merck) prior to the incubation of 0.5 mg whole cell lysate with Protein G Dynabeads (Invitrogen) in a total volume of 1 mL. To control for non-specific interactions we included several immunoprecipitation controls, including a protein G magnetic bead control in WT_MS_. An anti-FLAG antibody immunoprecipitation control in WT_MS_ and FLAG only expressing WT_MS_ was used to identify non-specific interactions of the anti-FLAG antibody. Additionally, an anti-human heavy chain seven myosin antibody was used as a non-specific antibody control in FLAG-tagged EccA_3_. WT_MS_, WT_MS_::pNFLAG::pTEK-4S-0X, WT_MS_::pNFLAG0615::pTEK-4S-0X whole cell lysates were incubated with 5 μg anti-FLAG antibody bound protein G Dynabeads. The WT_MS_::pNFLAG0615::pTEK-4S-0X whole cell lysate was also incubated with 5 μg anti-human myosin heavy chain 7 (MYH7, R&D systems) antibody to serve as a non-specific antibody control. IPs were incubated at 4 °C for 2 h, rotating. Beads were washed twice with IP buffer I (50 mM HEPES-KOH pH 7.5, 150 mM NaCl, 1 mM EDTA, 1% Triton X-100, 0.1% Sodium deoxycholate, 0.1% SDS) supplemented with protease inhibitors. Subsequently, beads were washed twice with IP buffer II (10 mM Tris-HCl pH 7.5, 1 mM EDTA, 500 mM NaCl, 0.5% IGEPAL® CA-630, 0.5% Sodium deoxycholate) before a final two washes with 50 mM Triethylammonium bicarbonate (TEAB). Beads were incubated with 1 μg trypsin in 150 μL 50 μM TEAB at 37 °C for 18 hours, shaking at 700 rpm. Recovered supernatants were dried in a Concentrator*plus* (Eppendorf), prior to resuspension in 50 μL 50 mM TEAB. Peptides were cleaned using the MagReSyn HILIC (ReSyn Biosciences) as stipulated by the manufacturer prior to storage of dried peptides at −80 °C.

### Mass spectrometry analysis

Mass spectrometry analyses of immunoprecipitated samples were done at the Proteomics and Metabolomics Facility at Colorado State University, United States of America. A total of 3 μL of peptides were purified and concentrated using an on-line enrichment column (Waters Symmetry Trap C18 100 Å, 5 μm, 180 μm ID x 20 mm column). Chromatographic separation was performed on a reverse phase nanospray column (Waters, Peptide BEH C18; 1.7 μm, 75 μm ID x 150 mm column, 45 °C) using a 60 minute gradient: 2–5% buffer B (0.1% formic acid in ACN) for 1 minute, followed by 5–40% buffer B over 54 minutes and 40–85% buffer B over 5 minutes at a flow rate of 350 nanoliters/min. Peptides were eluted directly into the Orbitrap Velos Pro (ThermoFisher Scientific) mass spectrometer equipped with a Nanospray Flex ion source (ThermoFisher Scientific) and spectra were collected over a m/z range of 400–2000 positive mode ionization. Ions with a charge state of +2 or + 3 were accepted for tandem mass spectrometry (MS/MS) using a dynamic exclusion limit of 2 MS/MS spectra of a given m/z value for 30 sec (exclusion duration of 90 sec). The instrument was operated in FT mode for MS detection (resolution of 60,000) and ion trap mode for MS/MS detection with a normalized collision energy set to 35%. Compound lists of spectra were generated using Xcalibur 3.0 software (ThermoFisher Scientific) with an S/N threshold of 1.5 and 1 scan/group.

### Identification of immunoprecipitated proteins

MaxQuant 1.6.7.0. was used to analyse mass spectrometry data using the *M. smegmatis* mc2 155 database (UP000000757) containing 8794 predicted protein sequences obtained from Uniprot, October 2014 [51, 52]. Carbamidomethyl cysteine was set as a fixed modification with four variable modifications, including oxidized methionine, the addition of glycine on lysine, serine and threonine residues, the addition of methylol and glycine on any histidine, asparagine, glutamine, tryptophan and tyrosine as well as the possible di-methylation of lysine and arginine residues. A total of two missed tryptic cleavages were allowed and proteins were identified with a minimum of 1 unique peptide per protein. The protein and peptide false discovery rate (FDR) was set at less than 0.01. Using the MaxQuant LFQ (MaxLFQ) algorithm, relative quantification was performed to obtain LFQ intensity values for identified protein groups. The “match between runs” algorithm was selected to detect peptides which were not selected for MS/MS analysis in the other anti-FLAG and control immunoprecipitation experiments. LFQ intensity data for identified proteins from the proteinGroups.txt file were used for statistical analyses using Perseus. All potential contaminants, proteins only identified by site and reverse hits were removed prior to log 2 transformation and filtering to remove proteins only identified with one unique peptide. Hierarchical clustering was done in Perseus to generate a principal component analysis plot using the default parameters to demonstrate that anti-FLAG IPs in WT_MS_::pNFLAG0615::pTEK-4S-0X clustered separate from control IPs. Proteins were deemed identified when present in at least two of the three anti-FLAG IPs. A list of high confidence proteins, that excluded proteins identified in the control immunoprecipitations, was constructed. Following the removal of high confidence proteins, a non-parametric Kruskal-Wallis test with an FDR of 0.05 and a Benjamini-Hochberg correction was used to identify low confidence proteins. Fold changes were calculated for each low confidence protein using the log2 transformed LFQ intensity data in relation to each of the control IP (Table S6).

MSMEG annotations, protein descriptions and gene ontology (GO) information was assigned using Uniprot (https://www.uniprot.org/) [52]. The database for annotation, visualization and integrated discovery (DAVID) (https://david.ncifcrf.gov/) was used for GO enrichment analysis of proteins identified in this study [22].

## Supplementary Information

**Additional file 1: Table S1.** Bacterial Strains. **Table S2.** Plasmids. **Table S3.** Primers. **Table S4.** Number of fluorescent foci at least 0.5 μm the from mid-cell of *M. smegmatis*. **Table S5.** High and Low confidence proteins. **Table S6.** Fold change of low confidence proteins. **Table S7.** Identifying characteristics of high and low confidence proteins. **Table S8.** Unique peptides of identified high and low confidence proteins. **Table S9.** Gene Ontology erichments of identified proteins.

**Additional file 2: Figure S1.** Detection of C-terminally GFP-tagged proteins in *M. smegmatis* and ESX-3 knockout *M. smegmatis*. **Figure S2.** Detection of N-terminally FLAG-tagged proteins in *M. smegmatis*. **Figure S3.** Principal component analysis of immunoprecipitations. **Figure S4.** Detection of C-terminally GFP-tagged proteins in *M. smegmatis* and ESX-3 knockout *M. smegmatis*. **Figure S5.** Detection of N-terminally FLAG-tagged proteins in *M. smegmatis*.

## Data Availability

The mass spectrometry data have been deposited to ProteomeXchange Consortium via the PRIDE partner repository (https://www.ebi.ac.uk/pride/archive) with the dataset identifier PXD018978 [53]. Data analysed for this study are included in this publication and its supplementary information files. Imaging flow cytometry data are available from the corresponding author upon request.

## Abbreviations

AP-MS: Affinity purification - mass spectrometry
ATP: Adenosine triphosphate
CAF: Central Analytical Facilities
ChIP-seq: Chromatin immunoprecipitation followed by sequencing
DAVID: Database for Annotation, Visualization and Integrated Discovery
DNA: Deoxyribonucleic acid
ESCRT: Endosomal sorting complex required for transport
FDR: False Discovery Rate
FLAG: DYKDDDDK polypeptide tag
GFP: Green fluorescent protein
GO: Gene Ontology
IP: Immunoprecipitation
LB: Luria-Bertani
LFQ: Label-free quantification
MRM: Multiple reaction monitoring
MS/MS: Tandem mass spectrometry
MYH7: Anti-human myosin heavy chain 7
PBS: Phosphate buffered saline
PCR: Polymerase Chain Reaction
PE: proline-glutamic acid
PEP: Posterior Error Probability Scores
PPE: proline-proline-glutamic acid
RNA-seq: RNA sequencing
Sec: General secretion pathway
SRM: Selected reaction monitoring
SRP: Signal recognition particle
TEAB: Triethylammonium bicarbonate
USA: United States of America
WT_MS_: Wild type *Mycobacterium smegmatis*
ΔESX-3_MS_: ESX-3 knock-out *M. smegmatis*
