# Supplementary material for: Localization of EccA3 at the growing pole in Mycobacterium smegmatis
Source: BMC Microbiol. 2022 May 19;22:140. doi: 10.1186/s12866-022-02554-6 (PMC9118679; doi:10.1186/s12866-022-02554-6)
Supplement: Supplementary file 2 — Additional file 2: Figure S1. Detection of C-terminally GFP-tagged proteins in M. smegmatis and ESX-3 knockout M. smegmatis. Figure S2. Detection of N-terminally FLAG-tagged proteins in M. smegmatis. Figure S3. Principal component analysis of immunoprecipitations. Figure S4. Detection of C-terminally GFP-tagged proteins in M. smegmatis and ESX-3 knockout M. smegmatis. Figure S5. Detection of N-terminally FLAG-tagged proteins in M. smegmatis. [file 12866_2022_2554_MOESM2_ESM.pdf]

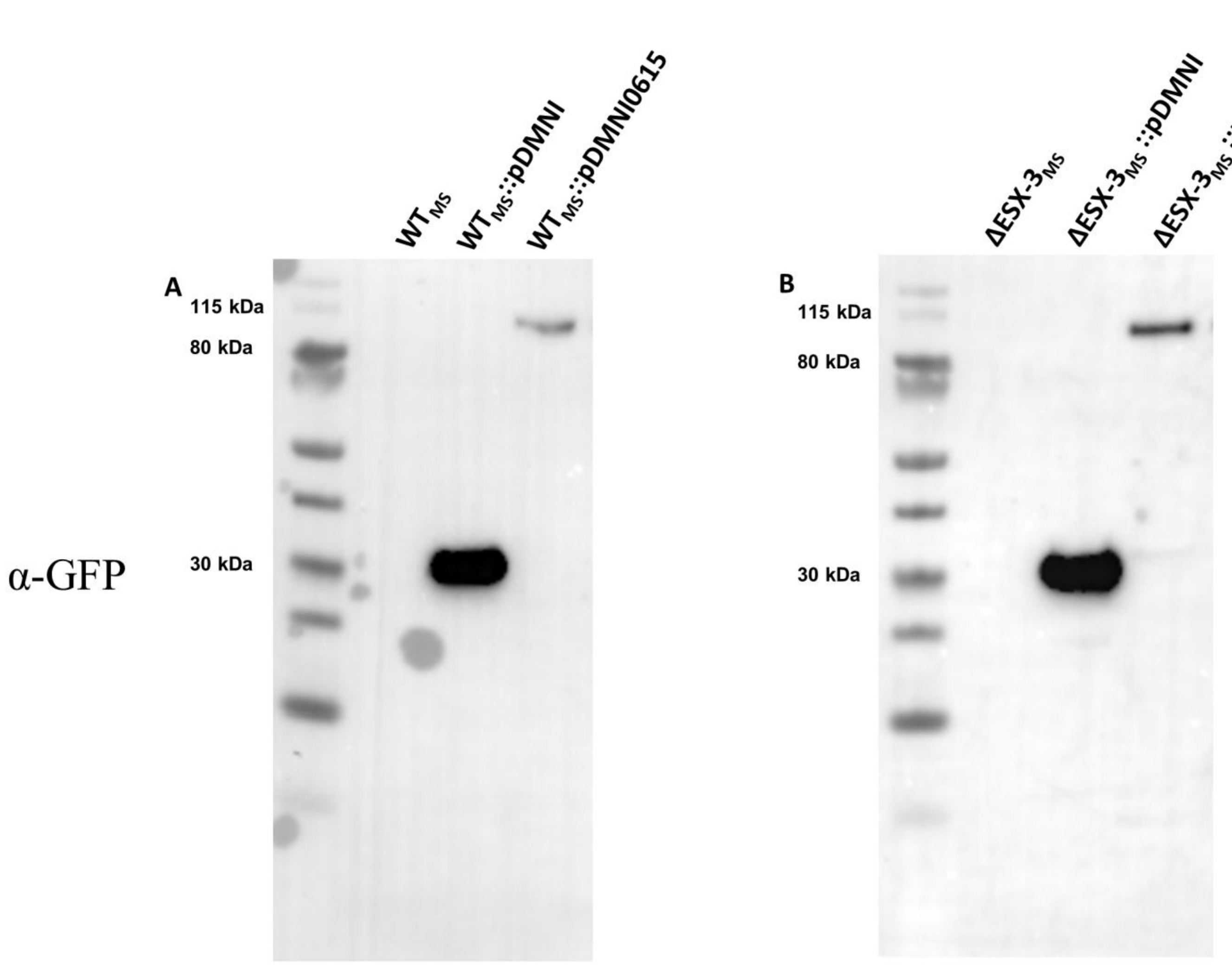

GFP	26.9 kDa
EccA3-GFP	93.2 kDa

Figure S1. Detection of C-terminally GFP-tagged proteins in M. smegmatis and ESX-3 knockout *M. smegmatis*. Western blotting was used to confirm the expression of GFP-tagged $EccA_3$ in WT_{MS} (A) and $\Delta ESX\text{-}3_{MS}$ (B) using an anti-GFP antibody. Full length GFP and $EccA_3$ was detected in both WT_{MS} and $\Delta ESX\text{-}3_{MS}$. The full-length blots are presented in Supplementary Figure S4.

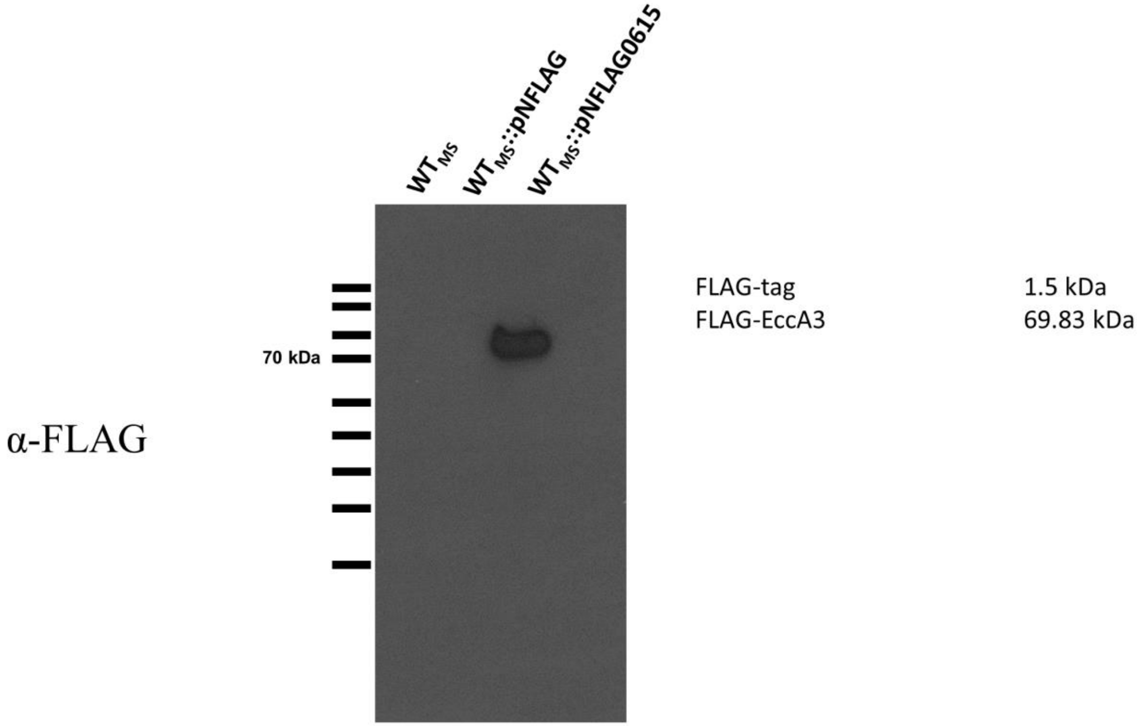

Figure S2. Detection of N-terminally FLAG-tagged proteins in *M. smegmatis*. Western blotting was used to confirm the expression of FLAG-tagged $EccA_3$ using an anti-FLAG antibody in WT_{MS}. The full-length blot is presented in Supplementary Figure S5.

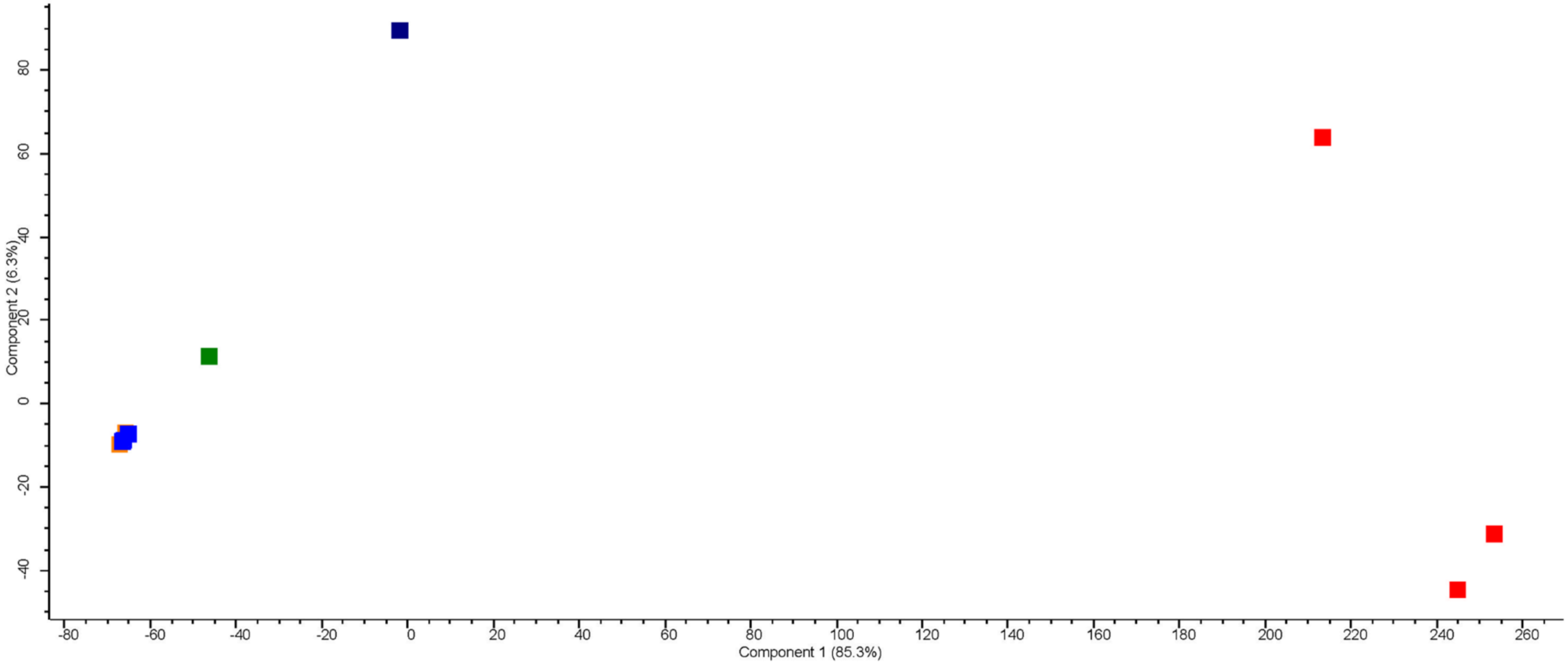

Figure S3. Principal component analysis of immunoprecipitations. Principal component analysis revealed separate clustering of anti-FLAG-EccA3 immunoprecipitations (red) from control immunoprecipitations for the 270 protein groups identified with at least two unique peptides. Protein G Dynabead control immunoprecipitations (blue), anti-FLAG control immunoprecipitations from WT_{MS} (orange) and WT_{MS}::pNFLAG (green) and anti-MYH7 control immunoprecipitations (dark blue) clustered on the left, separate from anti-FLAG-EccA3 immunoprecipitations on the right.

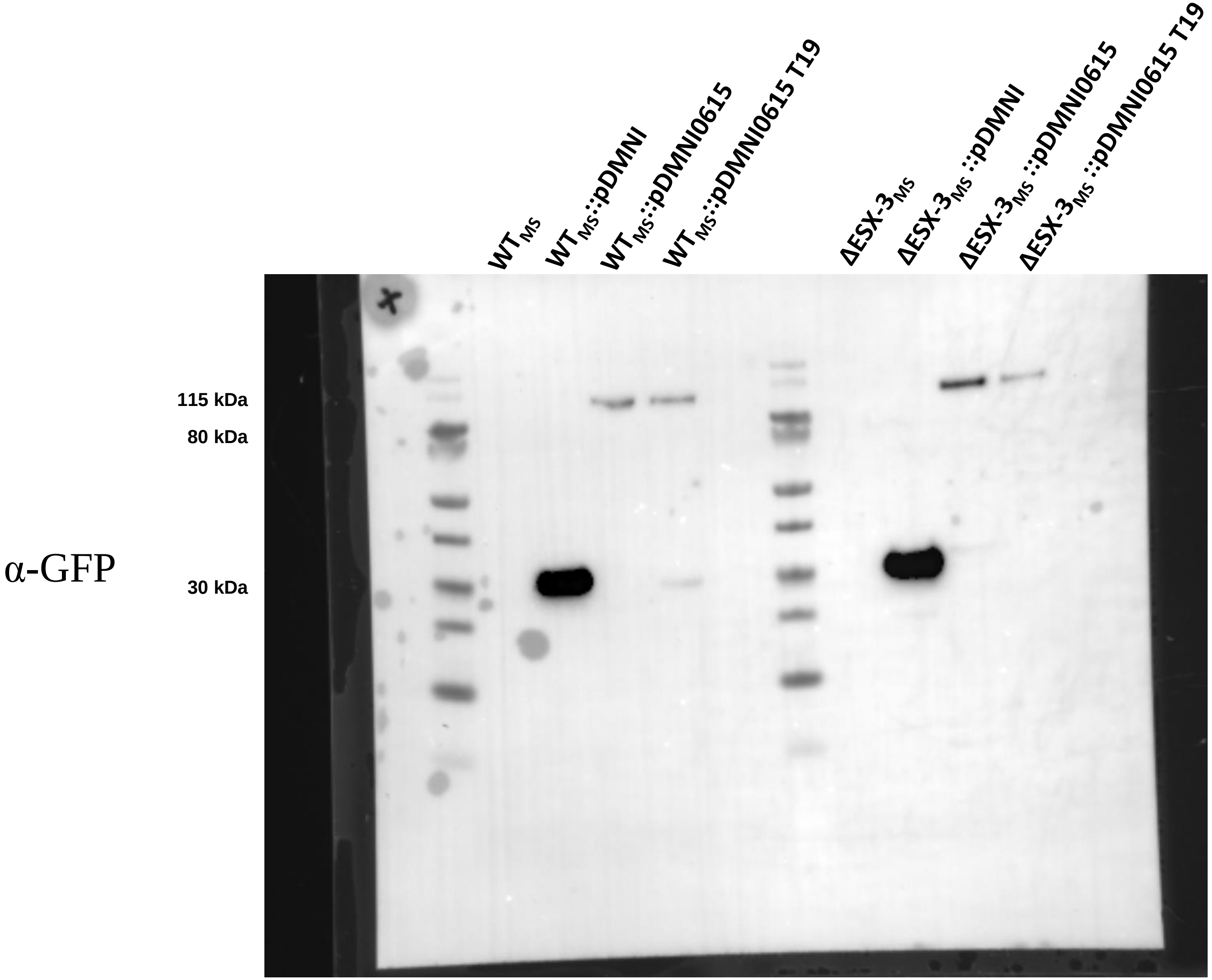

GFP	26.9 kDa
EccA3-GFP	93.2 kDa

WT_{MS}::pDMNI0615 T19 and ΔESX-3_{MS} ::pDMNI0615 T19 not discussed in this manuscript.

Figure S4. Detection of C-terminally GFP-tagged proteins in *M. smegmatis* and ESX-3 knockout *M. smegmatis*. Western blotting was used to confirm the expression of GFP-tagged $EccA_3$ in WT_{MS} and ΔESX-3_{MS} using an anti-GFP antibody. Full length GFP and $EccA_3$ was detected in both WT_{MS} and ΔESX-3_{MS}.

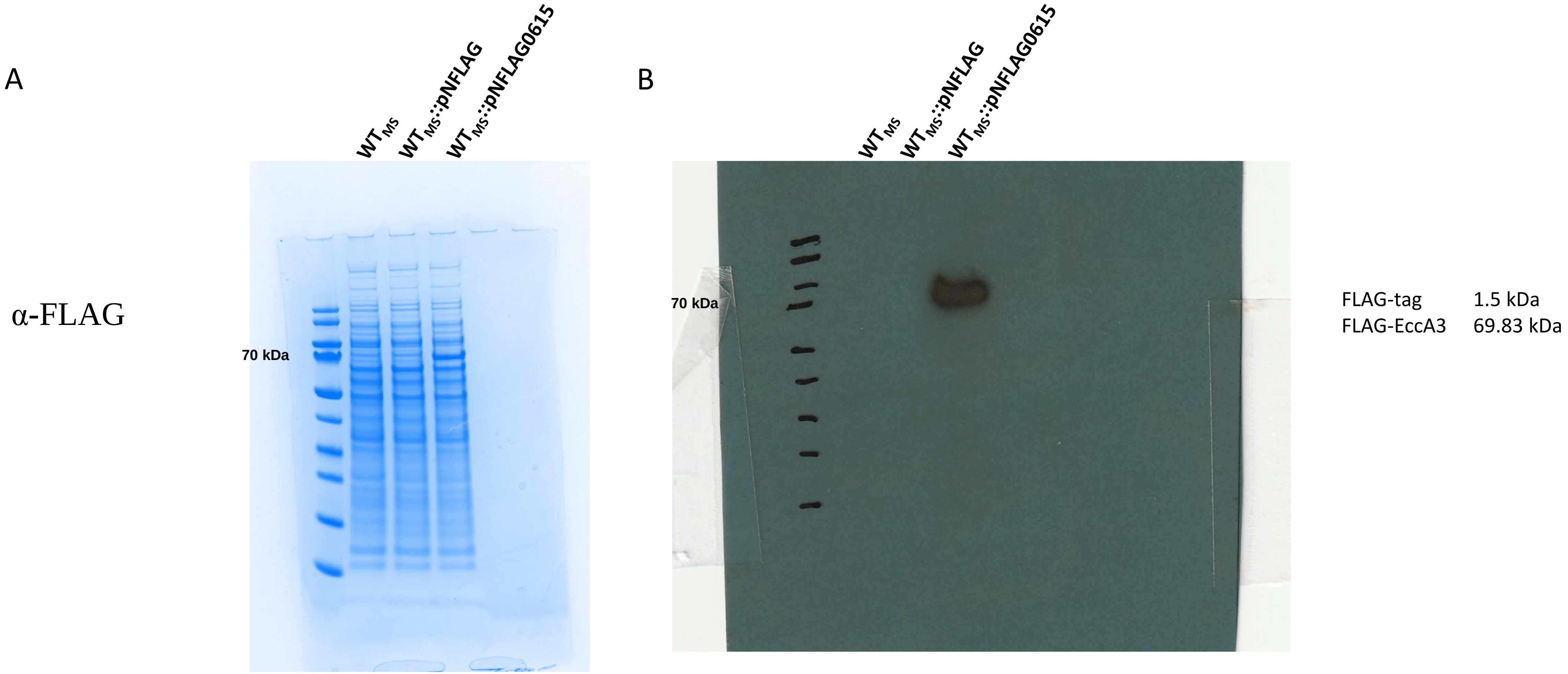

Figure S5. Detection of N-terminally FLAG-tagged proteins in *M. smegmatis*. A. SDS-PAGE of WT_{MS} and transformant strains WT_{MS}::pNFLAG and WT_{MS}::pNFLAG0615 cell lysates. **B.** Western blotting was used to confirm the expression of FLAG-tagged $EccA_3$ using an anti-FLAG antibody in WT_{MS}.
